# Hippocampal atrophy and memory dysfunction associated with physical inactivity in community‐dwelling elderly subjects: The Sefuri study

**DOI:** 10.1002/brb3.620

**Published:** 2016-12-29

**Authors:** Manabu Hashimoto, Yuko Araki, Yuki Takashima, Kohjiro Nogami, Akira Uchino, Takefumi Yuzuriha, Hiroshi Yao

**Affiliations:** ^1^ Center for Emotional and Behavioral DisordersNational Hospital Organization Hizen Psychiatric CenterSagaJapan; ^2^ Graduate School of Integrated Science and TechnologyShizuoka UniversityHamamatsuJapan; ^3^ Department of Diagnostic RadiologySaitama Medical University International Medical CenterSaitamaJapan

**Keywords:** Alzheimer's disease, dementia, magnetic resonance imaging, mild cognitive impairment, physical activity

## Abstract

**Background:**

Physical inactivity is one of the modifiable risk factors for hippocampal atrophy and Alzheimer's disease. We investigated the relationship between physical activity, hippocampal atrophy, and memory using structural equation modeling (SEM).

**Materials and Methods:**

We examined 213 community‐dwelling elderly subjects (99 men and 114 women with a mean age of 68.9 years) without dementia or clinically apparent depression. All participants underwent Mini‐Mental State Examination (MMSE) and Rivermead Behavioral Memory Test (RBMT). Physical activities were assessed with a structured questionnaire. We evaluated the degree of hippocampal atrophy (z‐score—referred to as ZAdvance hereafter), using a free software program—the voxel‐based specific regional analysis system for Alzheimer's disease (VSRAD) based on statistical parametric mapping 8 plus Diffeomorphic Anatomical Registration Through an Exponentiated Lie algebra.

**Results:**

Routine magnetic resonance imaging findings were as follows: silent brain infarction, *n *= 24 (11.3%); deep white matter lesions, *n *= 72 (33.8%); periventricular hyperintensities, *n *= 35 (16.4%); and cerebral microbleeds, *n *= 14 (6.6%). Path analysis based on SEM indicated that the direct paths from leisure‐time activity to hippocampal atrophy (β = −.18, *p *< .01) and from hippocampal atrophy to memory dysfunction (RBMT) (β = −.20, *p *< .01) were significant. Direct paths from “hippocampus” gray matter volume to RBMT and MMSE were highly significant, while direct paths from “whole brain” gray matter volume to RBMT and MMSE were not significant. The presented SEM model fit the data reasonably well.

**Conclusion:**

Based on the present SEM analysis, we found that hippocampal atrophy was associated with age and leisure‐time physical inactivity, and hippocampal atrophy appeared to cause memory dysfunction, although we are unable to infer a causal or temporal association between hippocampal atrophy and memory dysfunction from the present observational study.

## 1 Introduction

Old age is the single greatest risk factor for Alzheimer's disease (AD); physical inactivity, however, is one of the particularly strong predictors of incident AD (Beydoun et al., 2014; Yoshitake et al., 1995). Systematic reviews and meta‐analyses of prospective cohort studies provided compelling evidence that greater amounts of physical activity were associated with low‐to‐moderate risk reductions of dementia (Blondell, Hammersley‐Mather, & Veerman, 2014; Hamer & Chida, 2009; Sofi et al., 2011), whereas randomized clinical trials found inconsistent results that moderate‐intensity physical activity program, compared with the usual care program, provided modest improvement in cognition in subjects with memory problem (Lautenschlager et al., 2008) but not in sedentary older adults (Sink et al., 2015). Physical activity is defined as bodily activity that results in energy expenditure above resting levels; exercise refers to physical activity that is structured to meet specific fitness gains (Thomas, Dennis, Bandettini, & Johansen‐Berg, 2012). Higher levels of objective measures of total daily physical activity—activity energy expenditure assessed with doubly labeled water (Middleton et al., 2011) or actigraphy (Buchman et al., 2012)—were associated with a reduced risk of cognitive decline or AD.

Regular aerobic exercise can limit cognitive decline and the risk of dementia as a result of various neuroprotective mechanisms in the brain tissue especially in the temporal and prefrontal areas. Enhancement of trophic factor signaling induced by physical activity has been considered as the most popular hypothesis to explain the favorable effects of physical activity on cognition and brain structure; for example, myokines released by exercising muscle affect the expression of brain‐derived neurotrophic factor in the hippocampus (Erickson, Miller, & Roecklein, 2012; Phillips, Baktir, Srivatsan, & Salehi, 2014). Hippocampal atrophy is the most established structural AD imaging biomarker; structural MRI imaging is the most dynamic—among the major biomarkers of AD—in the prodromal phase of the disease (i.e., mild cognitive impairment [MCI]) (Jack, 2012; Petersen et al., 2009). Regional measures of hippocampal atrophy were the strongest predictors of progression from MCI to AD (Henneman et al., 2009). In this study, we examined associations among physical activity, structural MRI findings—gray matter atrophy in the whole brain and in the hippocampus, and memory function in nondemented elderly subjects.

## 2 Subjects and Methods

### 2.1 Study design and participants

This is a cross‐sectional observational investigation in community‐dwelling elderly people. Between 2010 and 2013, we examined memory function using Rivermead Behavioral Memory Test (RBMT) in 247 volunteers aged 58–94 years. They were living independently in the rural community of Sefuri village (Saga, Japan) without apparent dementia. A total of 34 subjects were excluded because of psychiatric disorders, including depression (*n *= 8), claustrophobia or contraindications to magnetic resonance imaging (MRI) (*n *= 5), a history of stroke (*n *= 9), brain tumor (*n *= 1), chronic subdural hematoma (*n *= 1), malignant neoplasm (*n *= 1), a history of head trauma (*n *= 4), chronic renal failure (*n *= 1), and insufficient clinical information (*n *= 4). Finally, we analyzed 213 subjects in this study (Table 1). The National Hospital Organization Hizen Psychiatric Center Institutional Review Board approved the study (approval number: 24–4), and written informed consent was obtained from all participants.

**Table 1:** Characteristics of the study population

Characteristics	Value (*n *= 213)
Age, mean (SD), years	68.9 (7.5)
Male sex, *n* (%)	99 (46.5)
Education, mean (SD), years	11.2 (2.0)
Mini‐Mental State Examination, mean (SD)	27.6 (2.2)
Rivermead Behavioral Memory Test, mean (SD)	19.1 (4.1)
Body mass index, mean (SD), kg/m^2^	23.4 (3.6)
Hypertension, *n* (%)	90 (42.3)
Systolic BP, mean (SD), mmHg	140.2 (19.3)
Diastolic BP, mean (SD), mmHg	82.2 (11.2)
Diabetes mellitus, *n* (%)	28 (13.1)
Hyperlipidemia, *n* (%)	70 (32.9)
Metabolic syndrome, *n* (%)	30 (14.1)
Alcohol, *n* (%)	86 (40.4)
Smoking, *n* (%)	19 (8.9)
Blood chemistry	
Albumin, mean (SD), g/l	44 (3)
FBG, mean (SD), mmol/L	5.69 (1.51)
HbA1c, mean (SD), %	5.7 (0.8)
LDL cholesterol, mean (SD), mmol/L	3.21 (0.88)
HDL cholesterol, mean (SD), mmol/L	1.76 (0.45)
eGFR, mean (S.D.), mL min^−1^ 1.73 m^−2^	74.5 (14.7)

BP, blood pressure; FBG, fasting blood glucose; LDL, low‐density lipoprotein; HDL, high‐density lipoprotein; eGFR, estimated glomerular filtration rate.

The participants underwent a structured clinical interview, biochemistry tests, and an electrocardiogram. Vascular risk factors were defined as previously described (Yao et al., 2015). Briefly, arterial hypertension was considered present if the subject had a history of repeated blood pressure recordings above 140/90 mmHg or the subject was being treated for hypertension. Diabetes mellitus was characterized by fasting plasma glucose greater than 7.77 mmol/L and/or HbA1c greater than 6.5% or a previous diagnosis of diabetes mellitus. Metabolic syndrome was defined according to the proposed criteria for metabolic syndrome in Japanese (Doi et al., 2009). We defined alcohol habit as consuming four drinks or more (one drink as 10 g of ethanol) per week. Smoking was defined as present if the subject smoked at least an average of 10 cigarettes per day.

### 2.2 Physical activity

Physical activity was assessed with a questionnaire modified from the Baecke questionnaire on habitual physical activity (Baecke, Burema, & Frijters, 1982) as previously described (Yao et al., 2015). The questionnaire consisted of three components: leisure‐time, work, and sport activities. Items concerning leisure‐time and work activities were coded on 5‐point scales. Leisure‐time index was assessed through four questions on comparison with others, sweat, sport, and walking. Work index was assessed through four questions on standing, walking, heavy loads, and sweat; other three original items (sitting, fatigue, and comparison with others) were excluded, because of the relatively weak associations (*r *< .6) of these questions with total score. Sport index was expressed as reported hours per week in each category multiplied by the metabolic equivalent of task (MET) (Ainsworth et al., 2014).

### 2.3 Cognition

Participants were tested individually in a quiet room. All participants underwent Mini‐Mental State Examination (MMSE) and RBMT. We administered neuropsychological tests according to the standard textbook (Strauss, Sherman, & Spreen, 2006), as previously described (Hashimoto, Takashima, Uchino, Yuzuriha, & Yao, 2014). Briefly, the purpose of MMSE is to screen for mental impairment particularly in the elderly. The RBMT consists of 11 subtests—first and second names, belonging, appointment, picture recognition, story (immediate, delay), face recognition, route (immediate, delay), messages (immediate, delay), orientation and date—with four parallel forms designed to identify memory deficits that might be encountered during daily living (Wilson, Cockburn, Baddeley, & Hiorns, 1989). The test does not adhere to any particular theoretical model of memory; instead, it attempts to mimic the demands made on memory by normal daily life. The standard profile score of RBMT yields scores between 0 and 24 (higher scores indicate better performance); subjects with standard profile score of RBMT less than 17 (the lowest quintile) were operationally defined as having memory dysfunction in the present analysis.

Each item of the Starkstein's apathy scale was quantified on a visual analog scale, where one end of a 60‐mm‐long line is “absolutely correct” and the other end is “completely wrong” as previously described (Yao et al., 2009).

### 2.4 Assessment of MRI findings

Imaging was performed on a 1.5T MRI scanner (Achieva, Philips, The Netherlands) using the T1‐ and T2‐weighted image, fluid‐attenuated inversion recovery (FLAIR), and T2*‐weighted images. The definitions and imaging methods were basically in line with the neuroimaging standards for research into small vessel disease (Wardlaw et al., 2013). Silent brain infarction (SBI) was shown as low signal intensities on T1‐weighted image, high signal intensities on T2‐weighted image, and their size was 3 mm or larger. We differentiated enlarged perivascular spaces from SBI on the basis of their location, shape, and size. The WMLs were defined as isointense with normal brain parenchyma on T1‐weighted images, and high signal intensity areas on T2‐weighted images. We used the validated rating scale of deep WMLs (DWMLs) by Fazekas et al. (1993): Grade 0, absent; Grade 1, punctate foci; Grade 2, beginning confluence of foci; and Grade 3, large confluent areas. For periventricular hyperintensities (PVHs), we determined the presence and severity (Grade 0, absent; Grade 1, pencil thin; and Grade 2, smooth halo lining) using FLAIR images. Two authors (H.Y. and A.U.), who were blinded to all clinical data, independently reviewed all scans.

**Figure 1:**
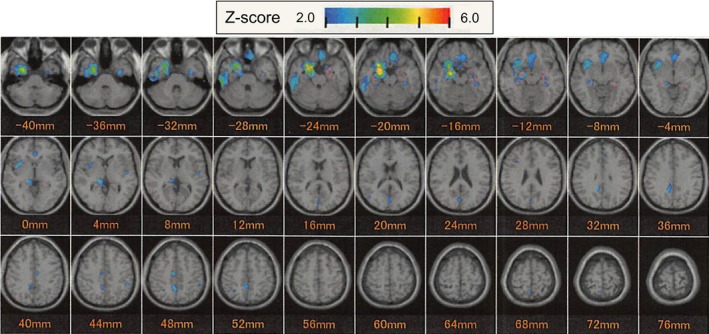
Representative images of the voxel‐based specific regional analysis system for Alzheimer's disease (VSRAD) advance version. Colored areas with z‐score of >2 are overlaid as significantly atrophied regions on tomographic sections of the standardized MRI template. In this case (No.15008, 79‐year‐old woman), the severity of atrophy obtained from the averaged positive values of *z*‐score in the hippocampus (ZAdvance) is 2.49, and the percentage rates of the coordinates with the *z*‐score >2 are 52.51% and 4.62% in the hippocampus and in the whole brain, respectively

We evaluated the degree of hippocampal atrophy, using a free software program—the voxel‐based specific regional analysis system for Alzheimer's disease (VSRAD) advance version (Matsuda et al., 2012) based on statistical parametric mapping 8 (SPM8) plus Diffeomorphic Anatomical Registration Through an Exponentiated Lie algebra (DARTEL) (Ashburner, 2007; Klein et al., 2009). To preserve gray matter volume within each voxel, Matsuda et al. (2012) modulated the images by the Jacobean determinants derived from the spatial normalization by DARTEL. We checked the segmentation process according to the VSRAD manual (http://www.vsrad.info/index2.html); qualified technicians (S. Morita and K. Kawakami) confirmed the good contrast between gray and white matter, no unacceptable irregularity in the images, no apparent artifacts, no unacceptable low intensities in T1‐weighted images, and no unacceptable ventricular enlargements. We determined the extent of atrophy as the averaged value of positive voxel‐by‐voxel z‐scores, where z‐score = ([control mean] – [individual value]) / (control SD) (i.e., the higher the value, the higher the extent of atrophy). We used three indicators—the severity of atrophy obtained from the averaged positive values of *z*‐score in the target (hippocampus) volume of interest (VOI) (hereafter referred to as ZAdvance), and the percentage rates of the coordinates with the *z*‐score exceeding the threshold value of 2.00 in the target (hippocampus) VOI and in the whole brain VOI—for characterizing atrophy of the hippocampus and the whole brain. Representative images are demonstrated in Figure 1.

### 2.5 Statistical analysis

Summary statistics of clinical variables were given as mean with standard deviation (SD). A significance level of 0.05 was used for statistical significance. The data were analyzed with the IBM SPSS Amos version 22.0 (SPSS Japan Inc., Tokyo, Japan). We used Pearson correlation coefficient to determine the presence or absence of a correlation between variables. Significant differences between mean were assessed by ANOVA and post hoc Bonferroni test for multiple comparisons. Multivariate analysis was carried out with the forward stepwise method of binary logistic regression analysis; before constructing structural equation modeling (SEM), we examined several different models or predictor variables, which cannot be tested with multiple regression analysis. We investigated the relationship between physical activity, hippocampal atrophy, and memory function using SEM (Haenlein & Kaplan, 2004). The SEM was described as path diagrams, where the square boxes represented measured observations, and circles represented latent constructs. Single‐headed arrows represented simple regression relationship, and double‐headed arrows represented correlations. We examined several indices of model fit for SEM analysis.

## 3 Results

**Figure 2:**
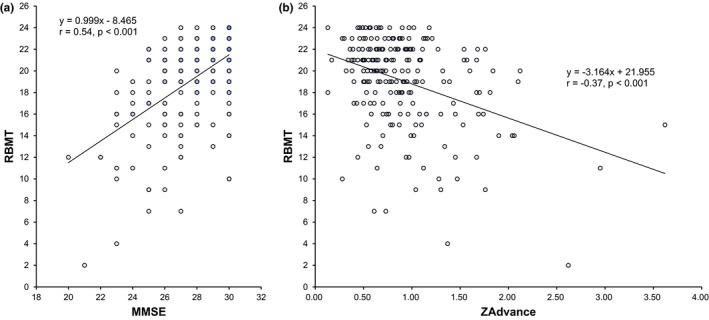
The relationship between Rivermead Behavioral Memory Test (RBMT) and Mini‐Mental State Examination (MMSE) (a) or between RBMT and hippocampal atrophy (ZAdvance) (b). The standard profile score of RBMT and MMSE was significantly correlated (Pearson correlation coefficient) (a). The higher ZAdvance score (i.e., hippocampal atrophy) was significantly correlated with the lower score of RBMT (b)

Characteristics of the study population are shown in Table 1. In the 213 subjects (99 men and 114 women with a mean age of 68.9 years), mean education (years of school) was 11.2 years. The mean of MMSE scores was 27.6 (SD 2.2), and the mean of RBMT (standard profile score) was 19.9 (SD 4.1); although the both scores were significantly correlated (Pearson correlation coefficient *r *= .54, *p *< .001), MMSE showed apparent ceiling effects in this nondemented population and seemed to have a low sensitivity for memory dysfunction (i.e., low RBMT scores) (Figure 2a). Silent brain infarction, DWMLs, PVHs, and cerebral microbleeds were detected in 24 (11.3%), 72 (33.8%), 35 (16.4%), and 14 (6.6%) subjects, respectively. Age, blood pressure, and blood chemistry panel were not different between the groups of different intensities in leisure‐time physical activities; body mass index (24.2 ± 3.9 [S.D.] kg/m^2^ vs. 22.6 ± 3.3 kg/m^2^, respectively) and waist circumference (86.2 ± 10.1 cm vs. 82.1 ± 9.0 cm, respectively) were significantly larger in high‐activity group (the highest tertile) compared with low‐activity group (the lowest tertile). Forty‐seven (22.1%) subjects reported engaging in sport activity (>4.0 MET•hour/week), of which walking was the most prevalent.

When possible confounders—including age, sex, education, hypertension, diabetes, and physical activities (leisure, work, and sport)—were entered into the binary logistic regression model (the forward stepwise method), the independent predictors of hippocampal atrophy, defined by the highest quintile of ZAdvance, were age (OR per 10 years 3.257, 95%CI 1.988–5.338, *p *< .001) and leisure‐time physical activity (OR per 1‐point increase 0.810, 95%CI 0.707–0.928. *p *= .002). In contrast, apathy was associated with less leisure‐time physical activity (OR = 0.594/100 points of apathy scale, 95% CI 0.416–0.848), but age, sex, education, and hippocampal atrophy did not enter into the equation with forward stepwise method of logistic regression analysis. The hippocampal atrophy (i.e., higher ZAdvance) was significantly correlated with the lower score of RBMT (*r *= −.37, *p *< .001) (Figure 2b) and MMSE (*r *= −.312, *p *< .001). The independent predictors of memory dysfunction, defined by the RBMT < 17 (48 of 213 [22.5%]), were age (OR per 10 years 2.173, 95%CI 1.340–3.524, *p *= .002) and hippocampal atrophy (OR per 1‐z‐score 3.756, 95%CI 1.663–8.484, *p *= .001).

**Figure 3:**
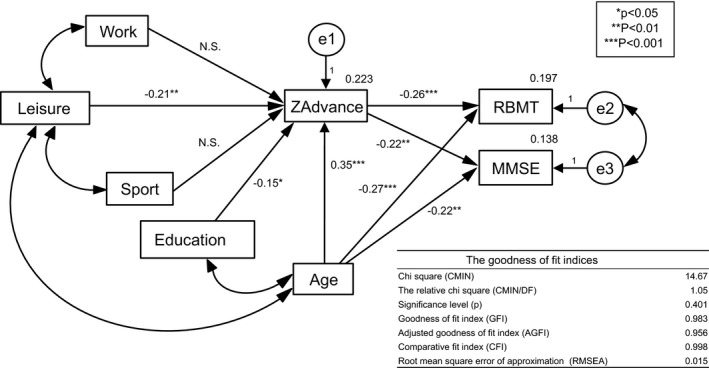
Structural equation modeling (SEM) of physical activity, hippocampal atrophy, and cognition. The direct path from leisure‐time physical activity to hippocampal atrophy (ZAdvance) was significant as was the direct paths from age and education to hippocampal atrophy. The direct paths from age and hippocampal atrophy to RBMT and MMSE were significant. The goodness‐of‐fit indices indicate that this presented model fit the data reasonably well

These findings mentioned above lead us to the hypothesis that physical inactivity would cause hippocampal atrophy and that hippocampal atrophy would cause memory dysfunction. Path analysis based on SEM indicated that the direct path from leisure‐time physical activity to hippocampal atrophy (ZAdvance) was significant (β = −.21, *p *< .01) as was the direct paths from age and education to hippocampal atrophy (Figure 3). However, the direct paths from work and sport activities to hippocampal atrophy were not significant. The direct paths from age and hippocampal atrophy to RBMT were highly significant (β = −.27 and −0.26, respectively, *p *< .001). The direct paths from age and hippocampal atrophy to MMSE were also significant. The measures of model fitness were as follows: relative chi‐square = 1.05, goodness‐of‐fit index = 0.983, adjusted goodness‐of‐fit index = 0.956, comparative fit index = 0.998, and root mean square error of approximation = 0.015. Thus, the presented model reasonably fit the data. We also presented the data as a form of multiple regression analysis predicting hippocampal atrophy in the Table S1.

In the same SEM, we analyzed the effects of atrophy of the hippocampus and the whole brain by replacing ZAdvance with the percentage rates of the coordinates with *z* values exceeding the threshold value of 2.00 in the target VOI (hippocampus) and in the whole brain (Table 2). The effects of “hippocampus” were essentially the same with those of ZAdvance; these two parameters were quite similar in that both indicate hippocampal atrophy by different definitions (see Methods). Direct paths from “hippocampus” gray matter volume to RBMT and MMSE were highly significant, while direct paths from “whole brain” gray matter volume to RBMT and MMSE were not significant. The effects of education on hippocampal atrophy were attenuated (0.05 < *p *< .1) in the analysis with the percentage rates of the coordinates with the *z*‐score exceeding the threshold value of 2.00 (Table 2).

**Table 2:** Relationship between independent and dependent variables with regard to gray matter atrophy

Independent variables → Dependent variables	Whole brain		Hippocampus	
	B	*p*	B	*p*
Age→Gray matter volume	0.385	<.001	0.299	<.001
Education→Gray matter volume	0.036	.628	−0.129	.079
Work→Gray matter volume	0.075	.245	0.006	.926
Leisure→Gray matter volume	−0.132	.058	−0.203	.003
Sport→Gray matter volume	−0.036	.597	0.052	.438
Gray matter volume→RBMT	−0.090	.186	−0.296	<.001
Gray matter volume→MMSE	−0.059	.398	−0.182	.008

B, standardized regression coefficient; RBMT, Rivermead Behavioral Memory Test; MMSE, Mini‐Mental State Examination.

## 4 Discussion

This study showed that hippocampal atrophy was associated with age, less education, and leisure‐time physical inactivity. The effects of education on hippocampal atrophy, however, were not robust in terms of inconsistent statistical significance among SEM results. Leisure‐time physical inactivity also tended to correlate with atrophy in the whole brain gray matter. Advanced age and hippocampal atrophy appeared to cause cognitive and memory dysfunction, while the relationship between atrophy in the whole brain gray matter and low cognitive function scores was not significant. Because hippocampal atrophy on neuroimaging is one of the plausible biomarkers for AD, the present results support the view that physical inactivity is one of the crucial risk factors for AD.

Several limitations of this study should be noted. First, because of the cross‐sectional and observational nature of this study, we are unable to infer a causal or temporal association between leisure‐time physical inactivity and hippocampal atrophy. Second, because our study primarily focused on hippocampus, the effects of physical activity on brain regions other than hippocampus were not completely clear. Third, the self‐report questionnaire for physical activity used in this study may not be accurate enough in capturing the type, intensity, and duration of the actual activity; comparisons of activity questionnaires with the doubly labeled water method showed generally low correlations (Erickson, Leckie, & Weinstein, 2014; Westerterp, 2009). The strength is that hippocampal atrophy was evaluated using the voxel‐based morphometry, which provided more precise spatial normalization to the template than the conventional algorithm; VSRAD advance version with DARTEL algorithm showed a high sensitivity (86.4%) and an extremely high specificity (97.5%) for discrimination of patients with very mild AD from healthy controls (Matsuda et al., 2012). An additional strength of this study includes the evaluation of memory function with RBMT in addition to the standard screening test (i.e., MMSE) in nondemented elderly subjects.

Physical activity and exercise are associated with volumes of specific brain regions including hippocampus as well as preserved cognitive function in healthy elderly subjects. Walking greater distance was associated with greater gray matter volume 9 years later in cognitively normal elderly subjects; this effect was predominant in prefrontal and temporal (i.e., hippocampus) brain regions (Erickson et al., 2010). Over the 18‐month follow‐up interval, hippocampus volume decreased only in the high risk (i.e., one or both APOE‐ε4 alleles) plus low physical activity group (Smith et al., 2014). Furthermore, physical activity reduced the abnormalities of white matter lesions; a higher level of physical activity was associated with higher fractional anisotropy, larger normal appearing white matter volume, and less white matter lesions—in addition to larger gray matter volume (Gow et al., 2012). The present findings were consistent with those of earlier studies that leisure‐time physical activity was associated with larger hippocampal volume and better memory function in nondemented elderly subjects. As Erickson et al. (2014) discussed, the positive association between higher physical activity and larger hippocampus volume may be one path by which physical activity reduces the risk for AD.

The benefits of aerobic activity or exercise on cognitive performance and brain structure are well documented. For example, Erickson et al. (2011) found that 1 year of aerobic exercise increased the volume of hippocampus by 2%, while the control stretching group displayed a 1.4% decrease in hippocampal volume. This study, however, showed that leisure‐time physical activity but not sport activity was significantly associated with preserved hippocampal volume; one possible explanation for the negative result for sport activity would be that only 47 of 213 subjects (22.1%) were engaged in sport activities, and the intensities were low—between strolling and walking at slow pace—in the half of the subjects with sport activities. This study also revealed that the beneficial effect of leisure‐time physical activity was not due to physical fitness, because high activity was associated with larger body mass index and waist circumference. Of note, even a low‐intensity “nonexercise” walking activity was associated with larger hippocampal volume among older women; therefore, increasing nonexercise or lifestyle physical activities rather than the intensity of activity per se may be important to preserve hippocampal volume (Varma, Chuang, Harris, Tan, & Carlson, 2015).

In conclusion, we found that hippocampal atrophy was associated with age, less education, and leisure‐time physical inactivity, and hippocampal atrophy was associated with memory dysfunction. Hence, leisure‐time physical activity could be a key modifiable factor against AD. Our previous study showed that one of the factors for leisure‐time physical inactivity was vascular depression or apathy in community‐dwelling healthy subjects (Yao et al., 2015). We regarded apathy as the quantitative reduction of self‐generated voluntary and purposeful (goal‐directed) behavior, suggesting the importance of behavior in terms of “voluntary” physical activity (Levy, 2012; Yao et al., 2009). Therefore, we would like to emphasize the critical role of motivation as an inherent demand for physical activity in the preventive strategy for dementia or AD.

## Conflict of Interests

None.
