# Supplementary material for: Hippocampal atrophy and memory dysfunction associated with physical inactivity in community‐dwelling elderly subjects: The Sefuri study
Source: Brain Behav. 2016 Dec 29;7(2):e00620. doi: 10.1002/brb3.620 (PMC5318373; doi:10.1002/brb3.620)
Supplement: Supplementary file 1 [file BRB3-7-e00620-s001.docx]

| Supplementary Table 1. Stepwise multiple regression analysis | | | |
| --- | --- | --- | --- |
| predicting hippocampal atrophy (ZAdvance) | | |  |
|  | B | 95%CI | p |
| Age, /10y | 0.230 | 0.138 ー 0.321 | <0.001 |
| Education, y | −0.029 | −0.050 ー −0.009 | 0.005 |
| Leisure | −0.034 | −0.067 ー −0.001 | 0.047 |
|  |  |  |  |
